# Neurovascular control following small muscle-mass exercise in humans

**DOI:** 10.14814/phy2.12289

**Published:** 2015-02-04

**Authors:** Tahisha M Buck, Steven A Romero, Matthew R Ely, Dylan C Sieck, Pedro M Abdala, John R Halliwill

**Affiliations:** Department of Human Physiology, University of OregonEugene, Oregon

**Keywords:** Dynamic knee extension, baroreflex, exercise, neurovascular, postexercise hypotension, sympathetic, transduction

## Abstract

Sustained postexercise vasodilation, which may be mediated at both a neural and vascular level, is seen in previously active skeletal muscle vascular beds following both large and small muscle-mass exercise. Blunted sympathetic vascular transduction and a downward resetting of the arterial baroreflex contribute to this vasodilation after cycling (large muscle-mass exercise), but it is unknown if these responses also contribute to sustained vasodilation following small muscle-mass exercise. This study aimed to determine if baroreflex sensitivity is altered, the baroreflex is reset, or if sympathetic vascular transduction is blunted following small muscle-mass exercise. Eleven healthy, college-aged subjects (five males, six females) completed one-leg dynamic knee-extension exercise for 1 h at 60% of peak power output. While cardiovagal baroreflex sensitivity was increased ∼23% postexercise relative to preexercise (*P* < 0.05), vascular and integrated baroreflex sensitivity were not altered following exercise (*P* = 0.31 and *P* = 0.48). The baroreflex did not exhibit resetting (*P* > 0.69), and there was no evidence of changes in vascular transduction following exercise (*P* = 0.73). In conclusion, and in contrast to large muscle-mass exercise, it appears that small muscle-mass exercise produces a sustained postexercise vasodilation that is largely independent of central changes in the baroreflex.

## Introduction

Arterial blood pressure is reduced following large muscle mass exercise such as treadmill running and cycling in humans (Halliwill et al. 2013). Associated with this postexercise hypotension, is a sustained postexercise vasodilation, which is due in part (perhaps 20%) to a downward resetting of the arterial baroreflex and a reduction in vascular responsiveness to sympathetic stimulation (Halliwill et al. 1996a), but mostly caused (perhaps 80%) by activation of histamine H_1_ and H_2_ receptors within the vascular bed of the previously exercised muscle (Lockwood et al. 2005; McCord and Halliwill 2006; McCord et al. 2006). Postexercise hypotension has been reported using a small muscle-mass exercise model of single-leg dynamic knee-extension exercise, albeit less consistently, and this hypotension is thought to be primarily mediated by sustained local vasodilation of the previously exercised quadriceps muscle (Barrett-O'Keefe et al. 2013). While it is possible there are also contributions from a reduction in sympathetic outflow or blunted sympathetic signal transduction following small muscle-mass exercise (as is true in whole-body exercise models), this has not been tested previously. Resetting of the baroreflex could conceivably be a function of the muscle mass involved, as it is linked to the activation of muscle afferent nerves (Chen and Bonham 2010). On the other hand, changes in transduction may be related to the relative metabolic state of the exercising muscle, and independent of the amount of muscle mass.

At minimum, the documentation of neurovascular control following single-leg exercise will clarify what aspects of the whole-body response such as baroreflex resetting and altered vascular transduction, if any, can be studied using this model. Further, an initial description of these characteristics may help generate novel hypothesis regarding the necessary and sufficient stimuli to evoke such changes in response to whole-body exercise.

Thus, this study was performed to determine (1) whether arterial baroreflex sensitivity is altered, (2) whether the arterial baroreflex resets, and (3) the potential contribution of a reduction in sympathetic neurovascular transduction to sustained postexercise vasodilation following single-leg dynamic knee-extension exercise, a model of small muscle-mass exercise. It was hypothesized that sympathetic neurovascular transduction would be blunted following exercise, but that baroreflex sensitivity would be unaltered and resetting would be absent following small muscle-mass exercise.

## Methods

This study was approved by the Institutional Review Board of the University of Oregon. Each subject gave written informed consent before participating in the study.

### Subjects

Eleven healthy, nonsmoking (exercise <3 h/week) college-aged men and women participated in the study (five males; six females). Based on the subjects' exercise habits over the previous 12 months and self-reported physical activity levels on two questionnaires (Baecke et al. 1982; Kohl et al. 1988), they were classified as recreationally active. Subjects refrained from taking any medications (except oral birth control pills), as well as from alcohol, vitamins, or doing any exercise 24 h before each study visit. Further, subjects refrained from consuming any caffeine 12 h before the study and complied with a 2-h fast before the study. All women had negative pregnancy tests before participation in both the screening and experimental days of the study, and were tested in the early follicular phase of their menstrual cycle or on placebo days if they were taking prescribed oral birth control in order to control for confounding effects of female sex hormones on autonomic-vascular regulation (Minson et al. 2000).

### Experimental design

Subjects came to the laboratory on two separate visits. The first visit consisted of a physical prescreening, familiarization to study procedures, and a graded dynamic knee-extension peak power exercise test. The carotid bifurcation was identified by ultrasound and the distance from the bifurcation to the angle of the mandible was measured in centimeters to ensure that the carotid neck collar would overlay the bifurcation. The peak exercise test was used to determine peak aerobic power output in the right leg of subjects, from which 60% of peak power output was calculated and used as the target power output during the experimental day as described previously (Barrett-O'Keefe et al. 2013).

On the experimental day, subjects performed dynamic knee-extension exercise on a custom built kicking ergometer controlled with custom software for 1 h at 60% of their peak power output at 45 kicks per minute. Subjects were instructed to actively extend at the knee against a bar and passively relax to the neutral position at 90° knee flexion to isolate quadriceps muscle group activation during exercise. Heart rate, arterial pressure, and femoral blood flow at rest and in response to neck pressure applied with a neck collar were measured before (Preexercise) and at 15, 30, 45, and 60 min postexercise in the supine position. Ambient temperature was controlled between 21 and 23°C throughout the experiment.

### Heart rate and arterial pressure

Arterial blood pressure was measured in the left arm using automated auscultation (Tango+, SunTech Medical, Raleigh, NC). The neck collar used for baroreflex testing was removed from the subject during measurements to avoid any possible carotid distension or compression by the neck collar during measurements. Heart rate was continuously monitored throughout the study day using a three lead electrocardiograph (Cardiocap/5 Critical Care Monitor, Datax-Ohmeda, GE Healthcare, Madison, WI). Beat-by-beat arterial blood pressure was continuously measured with a finger photoplethysmograph (Finometer; Finapres Medical Systems BV, Arnhem, the Netherlands), except during exercise. During the 60 min bout of dynamic knee-extension exercise, heart rate was continuously measured, and arterial pressure was measured at the arm every 10 min.

### Femoral blood flow

Femoral blood flow was measured with Doppler ultrasound (10 MHz linear-array vascular probe, GE Vingmed System 5, Horton, Norway) distal to the inguinal ligament, approximately 2–3 cm proximal to the bifurcation of the common femoral artery. A pressure cuff (Hokanson E20 Rapid Cuff Inflator, D. E. Hokanson, Inc., Bellevue, WA) was placed on both legs immediately distal to the patella, and inflated to 250 mmHg prior to measurements to ensure that blood flow measured in the femoral artery was indicative of blood flow to the thigh region and to eliminate blood flow though arteriovenous shunts in the feet. The entire width of the femoral artery was insonated with an angle of 60 degrees. Based on the knowledge that femoral artery diameter does not change during neck pressure (Pellinger and Halliwill 2007), femoral artery diameter was measured in triplicate during diastole using the built-in calipers of the ultrasound system at rest before each set of neck pressure trials. Real-time demodulation and analysis of the quadrature output of the forward and reverse Doppler frequencies from the ultrasound system was used to determine mean blood velocity, using an intensity-weighted algorithm and custom software. Subsequently, blood velocities were corrected for thin-beam error by using a correction factor as described recently (Buck et al. 2014), and multiplied by artery cross-sectional area to determine femoral blood flow. Femoral vascular conductance was calculated as the quotient of femoral blood flow and mean arterial pressure.

### Neck pressure

Neck pressure was administered by an external neck collar that enclosed the anterior two-thirds of the neck below the mandible and was controlled by a pressure controller (PPC-1000, Engineering Development Laboratory, Inc., Newport News, VA) and custom software. Neck pressure was applied at 50 mmHg to stimulate the carotid baroreceptors and elicit a baroreflex-mediated increase in sympathetic activity. Neck pressure at 50 mmHg has been shown previously to cause an increase in heart rate, mean arterial pressure, and a fall in femoral vascular conductance (Pellinger and Halliwill 2007). During the time before and between successive neck pressure applications, subjects breathed to an audio cue at 0.25 Hz to minimize respiratory sinus arrhythmia (Brown et al. 1993), but neck pressure was applied for 5 s during end-expiration breath holds. Neck pressure trials were excluded from analysis if the heart rate response was <4 beats per minute within the first 3–5 s of stimulus, as this is consistent with a failure of the neck collar application of pressure to the barosensory area. Hemodynamic responses to external neck pressure were averaged across at least three trials for each time point. Carotid sinus pressure was estimated as the difference between mean arterial pressure and neck collar pressure.

### Baroreflex assessments

Hemodynamic responses to neck pressure were used to assess baroreflex function as follows. (1) Cardiovagal baroreflex sensitivity, representative of the inhibition of parasympathetic neural control of the heart, was estimated as the peak heart rate response observed 1–7 s after the onset of neck pressure. (2) Vascular baroreflex sensitivity, representative of sympathetic stimulation of skeletal muscle vascular beds that have high sympathetic innervation, was estimated as the nadir femoral vascular conductance response observed in the rested leg 3–12 s after the onset of neck pressure. (3) Integrated baroreflex sensitivity, representative of the summated cardiac and vascular responses, was estimated as the peak mean arterial pressure response observed 2–9 s after the onset of neck pressure.

Resetting of the baroreflex was assessed by graphical comparison of the baroreflex relation between estimated carotid distending pressure and the respective end-organ responses (i.e., heart rate, femoral vascular conductance, and mean arterial pressure).

### Neurovascular transduction

Previous studies have assessed neurovascular transduction, or the extent to which a change in sympathetic nerve activity can produce a vasoconstriction, by using sympathoexcitatory maneuvers to produce concurrent increases in sympathetic nerve activity and vascular resistance (Halliwill et al. 1996a). In this study, comparison of the change in femoral vascular conductance in response to the neck pressure between the exercised and the rested leg was used as a proxy for assessing whether sympathetic neurovascular transduction was altered in the exercised limb.

### Statistical analysis

Preliminary analyses indicated no effect of sex on any outcome variable, so all subsequent analyses were performed with men and women combined as a single group. Variables were analyzed by mixed-model repeated-measures ANOVA with a priori contrasts of preexercise versus postexercise (SAS v9.2; SAS Institute, Inc., Cary, NC). When interaction effects (e.g., leg vs. time) were *P* < 0.10, we split the analysis to explore each leg's response independently relative to time. When main effects were *P* < 0.05, they were further explored by post hoc least square means testing. All values are expressed as means ± SEM when comparing group means across conditions, or as means ± SD when presenting subject characteristics.

## Results

Subject characteristics are shown in Table1 and are typical for a young healthy population. Carotid bifurcation distance was within the range of the inflatable portion of the neck collar in all subjects.

**Table 1:** Subject characteristics

	Mean±SD	Range
Age (years)	24 ± 5	18–27
Height (cm)	175 ± 13	162–188
Mass (kg)	70.0 ± 18.5	51.5–88.6
Body mass index (kg/m^2^)	22.5 ± 3.9	18.6–26.5
Baecke sport index (arbitrary units)	2.75 ± 0.73	2.20–3.48
Physical activity index (MET h per week)	25.4 ± 16.6	8.9–42.1
Left carotid bifurcation distance (cm)	3.8 ± 1.1	2.7–4.9
Right carotid bifurcation distance (cm)	4.3 ± 1.1	3.2–5.3

### Exercise

Target power output was 20 ± 8 W and subjects maintained 19 ± 8 W. Steady-state exercising heart rate was 100 ± 1 beats per min and mean arterial pressure was 101 ± 1 mmHg.

### Preexercise versus postexercise hemodynamics

Heart rate, arterial pressure, femoral blood flow, and femoral vascular conductance before and after exercise are presented in Table2. There were no changes in heart rate (*P* = 0.90) or mean arterial pressure (*P* = 0.39) from before to after exercise. Femoral blood flow in the exercised leg was elevated by 65 ± 19% following exercise (*P* < 0.05), but not in the rested leg (*P* = 0.21). Femoral vascular conductance in the exercised leg tended to be elevated by 64 ± 23% following exercise (*P* < 0.05), but not in the rested leg (*P* = 0.48) as shown in Table2.

**Table 2:** Hemodynamics

	Preexercise	Time postexercise (min)			
		15	30	45	60
Mean arterial pressure (mmHg)	79.4 ± 3.2	82.2 ± 3.4	82.2 ± 2.8	83.6 ± 3.6	82.9 ± 3.2
Heart rate (beats per min)	59.3 ± 3.2	59.6 ± 3.2	60.2 ± 3.7	59.9 ± 3.8	59.4 ± 3.5
Femoral blood flow (mL/min)					
Exercised leg	354 ± 51^†^	499 ± 32^*^	433 ± 42	415 ± 47	434 ± 44
Rested leg	324 ± 21	348 ± 33	356 ± 38	382 ± 41	375 ± 37
Femoral vascular conductance (mL/min/mmHg)					
Exercised leg	4.54 ± 0.66^†^	6.20 ± 0.48^*^	5.27 ± 0.46	5.17 ± 0.55	5.20 ± 0.48
Rested leg	4.13 ± 0.28	4.24 ± 0.35	4.39 ± 0.54	4.75 ± 0.56	4.47 ± 0.39

Values are means ± SEM; *n* = 11; ^†^*P* < 0.05 preexercise versus postexercise. ^*^P < 0.05 versus preexercise by post hoc analysis.

### Baroreflex assessments

**Figure 1:**
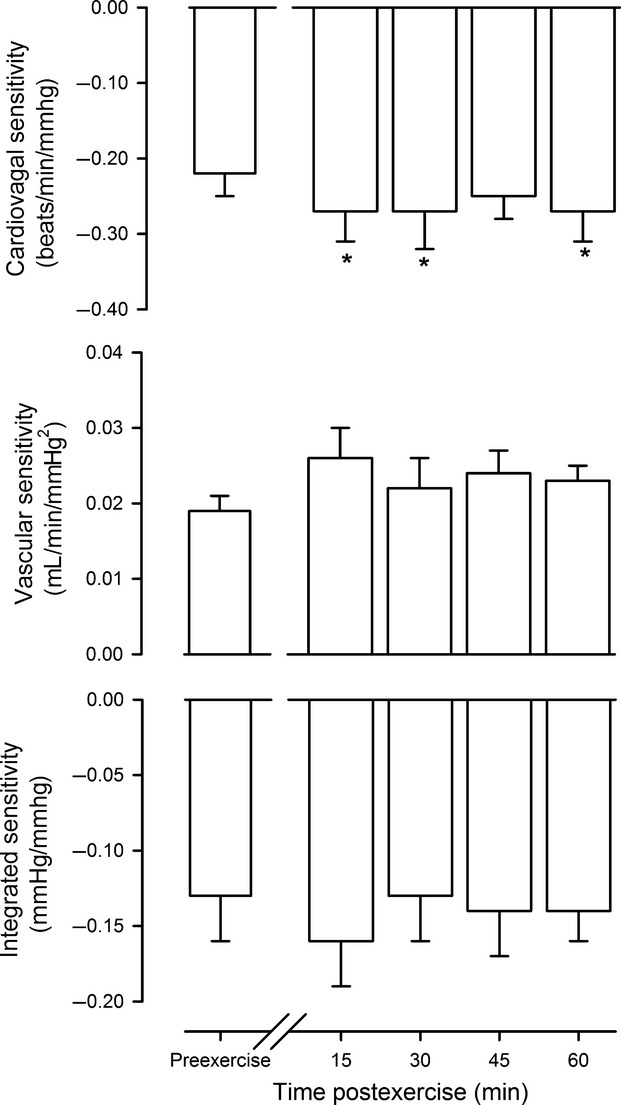
Baroreflex sensitivity. Cardiovagal sensitivity, vascular sensitivity, and integrated sensitivity of the baroreflex in response to neck pressure before (preexercise) and through 1 h postexercise. In this and subsequent figures, values are means ± SEM. *P < 0.05 versus preexercise.

Figure1 shows cardiovagal baroreflex sensitivity, vascular baroreflex sensitivity, and integrated baroreflex sensitivity before and after exercise. Postexercise cardiovagal baroreflex sensitivity was increased ∼23% relative to preexercise and had not recovered at 60 min (*P* < 0.05). In contrast, vascular baroreflex sensitivity and integrated baroreflex sensitivity were not different between preexercise and postexercise (*P* = 0.31 and *P* = 0.48).

**Figure 2:**
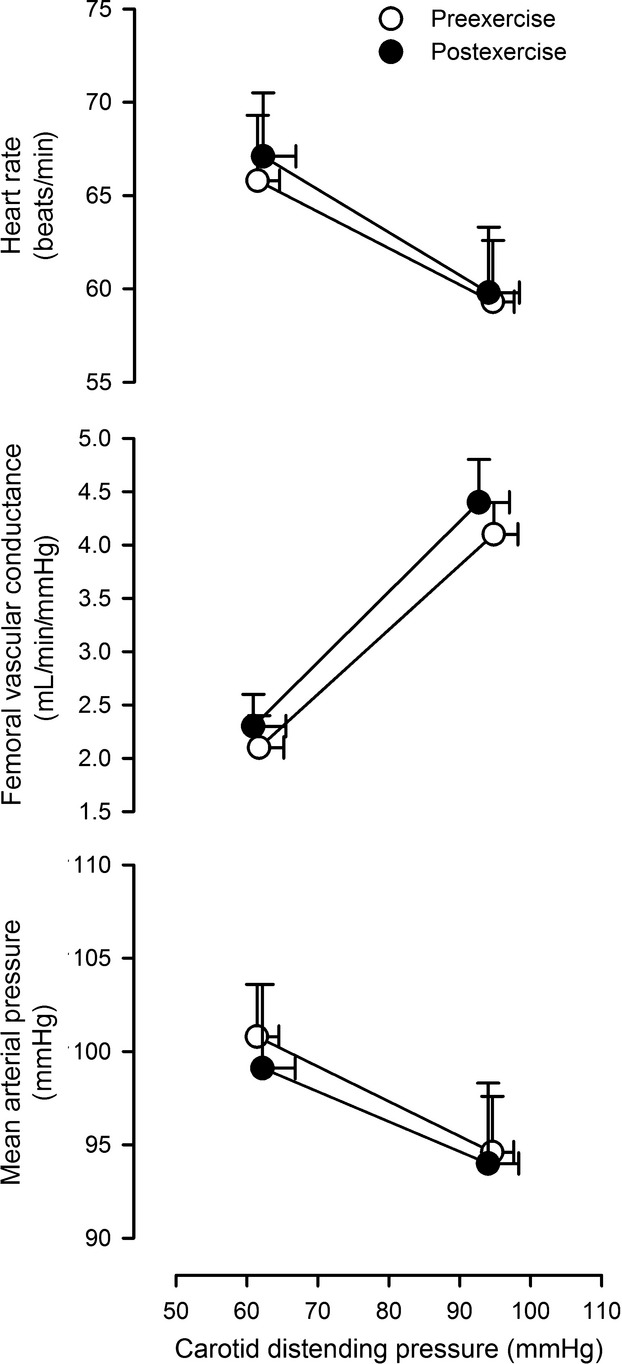
Baroreflex resetting. Comparison of the baroreflex relation between estimated carotid distending pressure and heart rate, femoral vascular conductance, and mean arterial pressure. Open circles denote preexercise; Closed circles denote average for all postexercise time-points.

Resetting of the baroreflex was assessed by graphical comparison of the baroreflex relation between estimated carotid distending pressure and the respective end-organ responses (i.e., heart rate, femoral vascular conductance, and mean arterial pressure) as shown in Figure2. There were no indications of resetting of the baroreflex, and analysis of the intercept of the linear regressions depicted in Figure2 which indicate an absence of any shift in relation to the *y*-axis intercept for heart rate (*P* = 0.87), femoral vascular conductance (*P* = 0.79), or mean arterial pressure (*P* = 0.69).

### Neurovascular transduction

**Figure 3:**
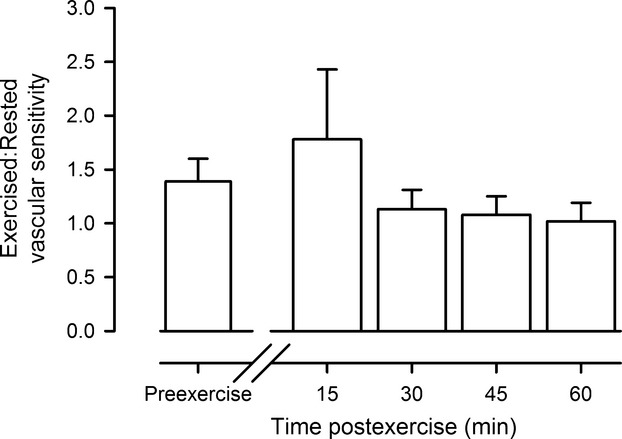
Neurovascular transduction. The ratio of vascular sensitivity in the exercised versus the rested leg before (preexercise) and through 1 h postexercise.

Comparison of the change in femoral vascular conductance in response to the neck pressure between the exercised and the rested leg, as shown in Figure3, was used as a proxy for assessing whether sympathetic neurovascular transduction was altered in the exercised limb. The ratio of vascular sensitivity between the exercised and the rested leg was unchanged from preexercise to postexercise (*P* = 0.73), indicating no change in vascular transduction in the exercised leg.

## Discussion

To the best of our knowledge, this is the first study to assess baroreflex sensitivity and test for the presence of resetting following dynamic small muscle-mass exercise. The main findings of this study are that cardiovagal baroreflex sensitivity is enhanced following dynamic knee-extension exercise, but there is no effect on sympathetic vascular baroreflex sensitivity, integrated baroreflex sensitivity, or neurovascular transduction. In addition, the baroreflex does not appear to be reset following dynamic knee-extension exercise of moderate intensity.

### Baroreflex sensitivity

Findings from the current study show that cardiovagal baroreflex sensitivity was augmented after exercise, while vascular baroreflex sensitivity was unchanged. In humans, the sensitivity of baroreflex control of sympathetic outflow is unaltered following large muscle-mass exercise (Halliwill et al. 1996a), but it appears blunted in rats (Kajekar et al. 2002; Miki et al. 2003). Thus, the lack of any change in vascular baroreflex sensitivity in the current study was not a foregone conclusion, but was unremarkable. As integrated baroreflex sensitivity is largely reflective of vascular responses, it is not surprising that it mirrored the vascular baroreflex and did not show changes following exercise.

In contrast to the sympathetic vasoconstrictor responses, cardiovagal baroreflex sensitivity following exercise has been more variable in studies following large muscle-mass exercise. Maximal exercise may briefly blunt cardiovagal baroreflex responses (Somers et al. 1985; Piepoli et al. 1993), yet can produce an increased cardiovagal baroreflex sensitivity later into recovery that can be observed up to 24 h later (Somers et al. 1985; Convertino and Adams 1991). Moderate-intensity exercise (e.g., 60-min of upright cycling at 60% VO_2_ peak) has been shown to augment cardiovagal baroreflex sensitivity (Halliwill et al. 1996b), reduce sensitivity (Terziotti et al. 2001; Willie et al. 2011), or have no effect on sensitivity (Halliwill et al. 1996a). Differences in methods of assessing baroreflex sensitivity as well as differences in exercise intensity or duration may account for the divergent results between studies.

### Baroreflex resetting

Although previous work has shown a resetting of the arterial baroreflex toward lower pressures after exercise in humans (Halliwill et al. 1996a) and in rats (Kajekar et al. 2002; Miki et al. 2003), the current findings provided no evidence to support the presence of resetting of the baroreflex after dynamic knee-extension exercise. In a series of studies reviewed in Chen & Bonham (2010), evidence has been provided that activation of group III and IV muscle afferent nerves during exercise cause the postexercise resetting by the process of internalization of neurokinin-1 receptors in the nucleus tractus solitarii. Based on this model, we suggest that either the duration or magnitude of muscle mass involvement (and therefore the magnitude of muscle afferent activation) during exercise directly contributes to postexercise resetting of the arterial baroreflex. Given this information, it is likely that the stimulus of exercise in the current study did not generate sufficient activation of muscle afferent nerves (too few nerves are recruited or the frequency of firing is too low) to produce enough neurokinin-1 receptor internalization to result in measureable resetting. Given the relative intensity of the exercise is similar to that in studies which generated resetting via whole-body exercise (e.g., cycling at 60% of VO_2_ peak for 1 h), it is likely that the muscle mass which is used is the primary factor which determines whether or not resetting will be generated. Alternatively, some evidence suggests that cardiac afferent nerves may play a role in inducing postexercise resetting of the baroreflex (Collins and DiCarlo 1993), and small muscle-mass exercise, such as in the current study, may generate significantly less activation of cardiac afferent nerves than whole-body exercise.

### Sympathetic neurovascular transduction

Postexercise hypotension and the associated sustained postexercise vasodilation may be mediated in part by a reduction in sympathetic neurovascular transduction after large muscle-mass exercise (Howard and DiCarlo 1992; Howard et al. 1992, 2000; Halliwill et al. 1996a). While both humans and animal models demonstrate a reduced vascular response, the mechanism may differ. Animal data show a reduction in postjunctional alpha-adrenergic receptor sensitivity (Rao et al. 2002), whereas human data show intact alpha-adrenergic receptor sensitivity after exercise (Halliwill et al. 2003) and suggest the presence of prejunctional inhibition of norepinephrine release. Both heterotrophic and autotrophic receptor inhibition may reduce norepinephrine release from sympathetic neurons, but it is not clear what neurotransmitter or receptors mediate such inhibition during recovery from exercise. Interestingly, the current study showed no change in neurovascular transduction postexercise. Thus, it appears that the amount of muscle mass involved in dynamic knee-extension exercise is not sufficient in magnitude to provoke changes in sympathetic neurovascular transduction.

### Perspectives

A single bout of aerobic exercise produces a postexercise hypotension associated with a sustained postexercise vasodilation of the previously exercised muscle. While we have determined some of the key pathways for these responses, the integrated nature of responses often makes mechanistic studies challenging to perform. In contrast to using whole-body exercise (i.e., large muscle-mass exercise), the single-leg dynamic knee-extension model may provide the ideal platform for exploring muscle-vascular interactions in the absence of central modulation by the baroreflex, changes in sympathetic nerve activity, and even changes in sympathetic neurovascular communication. While this was suggested in recent work from our laboratory (Barrett-O'Keefe et al. 2013), the current study put these notions to the test. It is likely that many additional studies will exploit this model for they study of recovery from exercise and sustained postexercise vasodilation. Likewise, studies directly comparing responses from small muscle-mass and large muscle-mass exercise may prove of value in testing hypotheses regarding the afferent signaling that is capable of producing a resetting of the arterial baroreflex in humans. In this context, Barrett-O'Keefe et al. reported a 27% elevation in vascular conductance at 1 h postexercise, which is comparable to the present study in which conductance was elevated 36% at 1 h postexercise. However, due to greater variability in the present study, this vasodilation was not a statistical elevation at 1 h. Also, Barrett-O'Keefe et al. noted a 3–4 mmHg reduction in arterial pressure after exercise, whereas pressure tended to rise postexercise in the present study. This suggests that any small changes in arterial pressure generated in this model may be masked by other influences such as circadian rhythms, subject heterogeneity, or subject's level of arousal due to the many measurements or paced breathing.

## Conclusion

This study provides novel evidence that arterial baroreflex is not reset and neurovascular transduction is not altered, yet cardiovagal baroreflex sensitivity may be increased following small muscle-mass exercise as exemplified by the single-leg dynamic knee-extension model. The absence of some of the changes observed following large muscle-mass exercise hint at possible mechanisms involved in baroreflex resetting in the context of postexercise hypotension.

## Conflict of Interest

None declared. This study was conducted by Tahisha M. Buck in part fulfillment of the requirements for the degree of Masters of Science at the University of Oregon.
